# An Accurate Deep Learning–Based System for Automatic Pill Identification: Model Development and Validation

**DOI:** 10.2196/41043

**Published:** 2023-01-13

**Authors:** Junyeong Heo, Youjin Kang, SangKeun Lee, Dong-Hwa Jeong, Kang-Min Kim

**Affiliations:** 1 Department of Mathematics Korea University Seoul Republic of Korea; 2 Visual Display Division Samsung Electronics Suwon Republic of Korea; 3 Department of Computer Science and Engineering Korea University Seoul Republic of Korea; 4 Department of Artificial Intelligence Korea University Seoul Republic of Korea; 5 Department of Artificial Intelligence The Catholic University of Korea Bucheon Republic of Korea; 6 Department of Data Science The Catholic University of Korea Bucheon Republic of Korea

**Keywords:** pill identification, pill retrieval, pill recognition, automatic pill search, deep learning, machine learning, character-level language model

## Abstract

**Background:**

Medication errors account for a large proportion of all medical errors. In most homes, patients take a variety of medications for a long period. However, medication errors frequently occur because patients often throw away the containers of their medications.

**Objective:**

We proposed a deep learning–based system for reducing medication errors by accurately identifying prescription pills. Given the pill images, our system located the pills in the respective pill databases in South Korea and the United States.

**Methods:**

We organized the system into a pill recognition step and pill retrieval step, and we applied deep learning models to train not only images of the pill but also imprinted characters. In the pill recognition step, there are 3 modules that recognize the 3 features of pills and their imprints separately and correct the recognized imprint to fit the actual data. We adopted image classification and text detection models for the feature and imprint recognition modules, respectively. In the imprint correction module, we introduced a language model for the first time in the pill identification system and proposed a novel coordinate encoding technique for effective correction in the language model. We identified pills using similarity scores of pill characteristics with those in the database.

**Results:**

We collected the open pill database from South Korea and the United States in May 2022. We used a total of 24,404 pill images in our experiments. The experimental results show that the predicted top-1 candidates achieve accuracy levels of 85.6% (South Korea) and 74.5% (United States) for the types of pills not trained on 2 different databases (South Korea and the United States). Furthermore, the predicted top-1 candidate accuracy of our system was 78% with consumer-granted images, which was achieved by training only 1 image per pill. The results demonstrate that our system could identify and retrieve new pills without additional model updates. Finally, we confirmed through an ablation study that the language model that we emphasized significantly improves the pill identification ability of the system.

**Conclusions:**

Our study proposes the possibility of reducing medical errors by showing that the introduction of artificial intelligence can identify numerous pills with high precision in real time. Our study suggests that the proposed system can reduce patients’ misuse of medications and help medical staff focus on higher-level tasks by simplifying time-consuming lower-level tasks such as pill identification.

## Introduction

### Background

It is important to identify the type of medication to avoid medical errors because it directly influences patients’ health and causes enormous losses. The Organisation for Economic Co-operation and Development (OECD) reported that 18.3% of adverse events in patients are medication errors [1]. The misuse of medications is a critical issue for patients, resulting in adverse medication effects such as injuries and complications [2]. In addition, the estimated health care cost caused by adverse medication events was more than US $76 billion in 2014 and has had an upward tendency yearly [3,4]. Nowadays, the types of medications are highly diverse owing to the rapid development of new medications and increased international trade of medications. As a result, it burdens medical staff because they should find pills manually from the pill database whenever patients ask for identifying their prescribed medications. This is because patients often discard their medication’s container that includes the prescription. In addition, medication errors can be caused by polypharmacy such as prescribing cascade and poor medication reconciliation [5]. To alleviate these errors, many countries have recently developed computerized medication systems that use information technology to identify medications and recognize the interactions between medications. For example, in South Korea, the Ministry of Food and Drug Safety (MFDS) has provided specifications for medications, including ingredients, images, and precautions. On the basis of their database, which is updated when a new pill is approved and enrolled, medical staff can check for potential adverse medication events, and patients can also identify medications [6-8].

However, it is necessary to develop automated systems because the current computerized medication systems that require the passive input of surface information have disadvantages in terms of use. Although the current computerized medication systems are helpful in preventing the misuse of medications, they require pharmaceutical expertise and significant labor to search for pills. This is largely attributed to the fact that users such as medical staff and patients must manually enter the exact names or properties of the medications. Previous studies have shown that pharmacists at university hospitals have spent an average of approximately 20 hours a month identifying medication, but approximately 25% of the medications were not identifiable in the conventional system [9,10]. The results were similar when expanding the study scope to a city [11]. They pointed out that this result was not only because of the broken pills but also because it was difficult for the medical staff to carefully identify pills using the conventional system. Although pills are the most widely used medication forms owing to their convenience of use and storage, it is difficult to identify the information on pills (eg, imprints) owing to their small size. Thus, although systems for identifying pills have been established in most countries, the preceding results highlight the limitations of the current pill search systems, despite their importance in the clinical field. In addition, it is very difficult for patients—who have no medical expertise—to identify pills while taking multiple doses of different medications in their daily lives. Therefore, many attempts have been made to develop automated pill search systems. These systems aim to help medical staff by mitigating the lower-level workload of pill identification and patients by providing appropriate information to avoid the intake of wrong pills. Although there are several studies aimed at developing automated pill identification systems, most of them have focused on recognizing characters imprinted on pill surfaces [12-16]. However, because the imprints on pills are small and contain abbreviations, it is difficult to recognize them quickly and accurately. Therefore, it is necessary to develop a fast and accurate automatic system that identifies pills by recognizing the imprinted characters with high accuracy.

### Prior Work

Surveys on medication errors and misuse highlighted the need for automatic, accurate, and rapid pill identification [4,17,18]. Recently, several studies have proposed artificial intelligence (AI)–based pill recognition systems. These approaches can be a promising research direction because the performance of medical staff in pill identification was improved with the assistance of an AI-based system. Cho et al [19] discussed a method for identifying pills using image processing methods. In 2016, the Pill Image Recognition Challenge was held in the United States, and 7000 images were provided as a data set for 1000 pills. Pill recognition models using this data set were actively studied, even after the conclusion of the competition [20]. Zeng et al [21] won the competition by proposing a model that utilized a triple network to distinguish between similar pills. Larios Delgado et al [22] proposed a model that utilizes a deep convolutional neural network (CNN) and compared it with various image classification models pretrained on ImageNet. Chang et al [23] proposed pill classification devices using a feature pyramid network with a 50-layer Residual Network (ResNet) as the backbone. However, they focused on real-time object detection, and the accuracy of pill identification was not a major consideration. The model proposed by Wong et al [16] used a GoogLeNet Inception network [24] based on a CNN and input images captured by the authors under various conditions. The model in the study by Wang et al [25] used AlexNet [26] and focused on the geometric transformation of pills in images. In previous studies on the identification of pills, all pill types in the test data set were included in the training data set with different images. Consequently, the models proposed in previous studies have 2 critical issues: (1) they suffer from a lack of generalizability in data sets that differ from their training data set, and (2) they find it difficult to identify newly approved pills.

Previous studies have commonly preprocessed pill images and then used image classification models to learn the surface area information of pills to perform classification tasks within a data set. Despite their diversity, several pills share limited information on appearance features (ie, shape, color, and form). For example, many pills take the form of white circular tablets. This reduces the accuracy of pill identification and is a challenging factor in pill search models. Therefore, to increase the accuracy of pill identification, information on the characters imprinted on the pills can play a critical role. Researchers have also been aware of the importance of these characters and have attempted to solve this problem through image preprocessing. Traditional pill search models [21,22] rely only on image classification methodologies, even if imprinted data are preprocessed through grayscale transformations, lighting control, or noise removal in images. However, it is difficult to distinguish similar character sequences such as “MIO” and “M10” because they are likely to be recognized as the same image when the shapes of the characters are similar. In addition, previous methods [13,17-23,25] impose the burden of updating the model whenever a new drug is developed because they only include registered pills in the evaluation data set, which leads to poor generalization. Meanwhile, there is a study that considered the characters imprinted on pills and used national drug codes for the identification of pills [27]. However, because the recognition of pill imprints depends solely on the image classification model (ResNet-18), there is a high probability of incorrect character recognition, which can lead to the incorrect prediction of drug codes, as in previous studies.

### Goal of This Study

Our method considers the imprinted characters on pills as crucial information for pill identification. We adopted a character-level language model and convolutional networks for recognizing other features (ie, shape, color, and form). In addition, we divided the types of pills in the training and evaluation data sets to improve generalizability and thus the identification of new pills. We overcame the limitations of the existing pill search models by designing a system that focuses on imprinted characters. First, the object detection model *You Only Look Once* (YOLO) [28] version 5 [29] was used to learn the locations and types of imprinted characters in a pill image. Next, the object recognition model (ie, ResNet-32) was used to learn the shape, color, and form of the pill [30]. In addition, we drew inspiration from the natural language processing field and considered the features of pills as context to learn the imprinted characters on pills in units of alphabet and number. In this study, the appearance of the pill (ie, shape, color, and form) is defined as *features*, and features and the imprinted characters are collectively referred to as *characteristics*.

The character-level language model receives the characters detected by the object detection model and modifies them to match the shape and order of the characters on the pill. For example, when it receives an input of “MOI,” it predicts the next letter after “M” based on the appearance of the pill (ie, features) and corrects it. We separately trained an imprint recognition model that extracts imprints from images of pills and an imprint correction model that corrects characters based on the context of recurrent neural networks (RNNs) [31-35]. Moreover, the features of pills were utilized as a context of imprints in the character-level language model. Consequently, a successful pill identification ability was established without complicated preprocessing of images for imprint identification. We noted that the character correction module is important because pill images may contain important information such as medicinal ingredients, amounts of ingredients, and pharmaceutical company names. We considered a total 24,404 pills: 20,517 (84.07%) pills using the data provided by the MFDS in South Korea and 3887 (15.93%) pill samples from the National Library of Medicine (NLM) [20] database in the United States. The main contributions of this study are described in Textbox 1.

**A summary of the main contributions and results of this study.**

- We proposed a pill identification system based on a deep learning approach. The proposed model extracts 3 features (ie, shape, color, and form) and imprinted characters from a given pill image and retrieves results from a database, even for pills that are not in the training data set.
- We incorporated a character-level language model into the proposed pill identification system. To the best of our knowledge, we are the first to introduce a language model into a pill identification system. Moreover, we proposed a novel coordinate encoding technique for imprinted characters on pills. Our model extracts the features of pills and uses them for classification as well as for context in the language model to improve the performance.
- We confirmed that our model is robust to newly approved pills and generalizable on 2 different databases: one from the Ministry of Food and Drug Safety (MFDS; South Korea) and the other from the National Library of Medicine (NLM; the United States). Our model is also applicable to consumers because it can evaluate consumer images.
- We evaluated the proposed system with the MFDS and NLM data sets. Our system achieved 85.65% and 74.46% accuracy of the top-1 candidate for the types of pills not used in the training for the MFDS and NLM data sets, respectively. Moreover, our model (78%) outperformed the baseline (76.9%) in evaluating consumer images.

### Organization

The remainder of this paper has been organized as follows. In the next section, we introduce and analyze pill databases. We then discuss the overall process of the proposed system and the implementation of each module in the recognition unit and search units. In the *Results* section, the experimental setups, analyses, and results are presented. We have evaluated the proposed system on the types of pills that were not used in the training and demonstrated its ability to identify newly approved pills using reference images in the MFDS and NLM databases. In addition to the consumer images with varying lighting conditions in the NLM database, we have highlighted the effectiveness of the proposed system by comparing it with a state-of-the-art model. Finally, we discuss the results and conclusions.

## Methods

### Database

**Figure 1:**
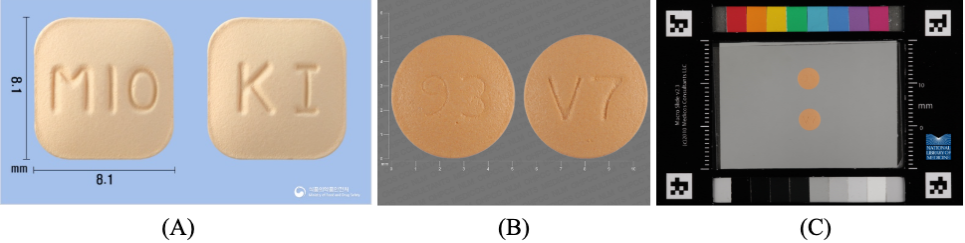
(A) An example of the reference image from the Ministry of Food and Drug Safety database is shown. (B) An example of the reference image from the National Library of Medicine database is shown. (C) An example of the consumer image from the National Library of Medicine database is shown.

In total, 2 data sets of pill images were utilized in this study. The database provided by the MFDS, which included sample images of 20,517 (N=24,404, 84.07%) pills, was used for the experiments. In this database, each pill has one reference image, and each image includes both the front and rear photos of the pill (Figure 1A). Of 20,517, we separated the samples into 8000 (38.99%) samples for a training data set and 12,517 (61.01%) samples for a test data set. To evaluate the system’s performance, we constructed the test data set with pills that were completely different from those in the training data set. We adopted this setup by assuming that new pills are updated in the database. Another database provided by the NLM in the United States, which is widely used in pill recognition tasks, was also adopted for evaluating whether the system can be applied to different data sets. This database includes various forms of images of pills. As a result, images from the NLM database, which consist of both faces of pills without additional information, for each of the 15.93% (3887/24,404) of pills were used for the experiments (Figure 1B). We fine-tuned the model using 25.73% (1000/3887) of reference images, and the remaining 74.27% (2887/3887) of reference images were used for the evaluation. Furthermore, we also evaluated the model with consumer-grade pill images given by the NLM to examine its robustness in terms of more challenging inference and generalization ability. In the NLM database, the size, position, and lighting of the consumer-grade pill images were not adjusted (Figure 1C) In the evaluation using consumer-grade pill images (hereinafter consumer images), the model was fine-tuned with randomly chosen 25.73% (1000/3887) of reference images. The remaining 74.27% (2887/3887) of pills were not used in the training data set but were used in the test data set. It should be noted that the 25.73% (1000/3887) of pill species in the reference image samples in the training data set are the same as the 25.73% (1000/3887) of pill species in the test sets but the images are different. In this work, we consider only uppercase characters, except for “mg” (ie, milligram), for analysis to improve the identification of characters. We discuss this issue in the limitations section.

In the MFDS database, the shapes can be classified into 10 types: round, oblong, oval, triangle, square, diamond, pentagon, hexagon, octagon, and others. “Others” includes unusual shapes such as semicircle, adjacent circle, bullet shape, rectangular shape with a concave center, heart shape, etc. As for colors, there were 16 categories: white, yellow, orange, pink, red, brown, light green, green, cyan, blue, navy, purple, gray, black, violet, and transparent. Finally, the forms included were tablets and capsules (Multimedia Appendix 1). We found that white circular tablets were the most prominent in the data set. In the NLM database, the most common shapes of pills were circular (1795/3887, 46.18%), followed by oval (1361/3887, 35.01%) and oblong (601/3887, 15.46%). There were <1% of the following shapes: square, triangle, pentagon, hexagon, octagon, diamond, semicircle, rectangle, and “others.” In the training data set for fine-tuning, we mapped the rectangular shape to square and others (eg, tear, double circle, or trapezoid) to “others.” The most common color was white (1643/3887, 42.27%), followed by yellow (493/3887, 12.68%), pink (347/3887, 8.93%), orange (336/3887, 8.64%), blue (328/3887, 8.44%), brown (224/3887, 5.76%), and green (206/3887, 5.53%). Less than 5% of the pills were red, purple, gray, turquoise, or black in color. The proportions of capsules and tablets were 15% and 85%, respectively.

### Model Architecture

**Figure 2:**
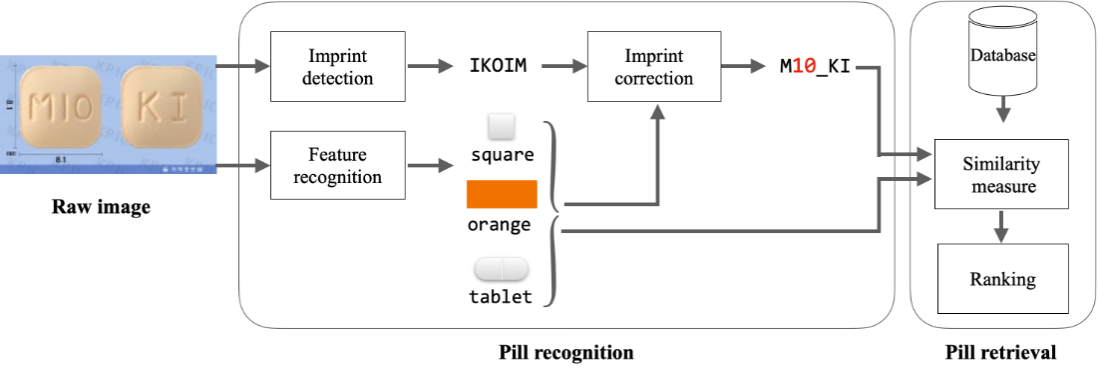
A pipeline of our system to recognize the pills’ characteristics and retrieve from database. IKOIM denotes an example of detected imprint from the raw image and M10_KI denotes an example of corrected imprint from IKOIM.

The proposed framework is illustrated in Figure 2. In the pill recognition step, a pill image is fed into the object detection model YOLOv5 [28,29] and image classification model ResNet-32 [30]. YOLOv5 extracts characters and their coordinates from the image, whereas ResNet-32 recognizes and classifies the pill’s shape, color, and form. The shape, color, form, and characters with their coordinates are then used as inputs for the RNN-based character-level language model [31-35]. In the character-level language model, the input characters are corrected using the coordinates of the characters, with the shape, color, and form of the pills acting as contexts. In the pill retrieval step, the similarity is calculated and then ranked by comparing the features (ie, shape, color, and form) extracted from ResNet-32 and imprints corrected from the RNN with the characteristics (ie, features and imprints) of the pills in the databases.

### Pill Recognition

#### Imprint Detection

In this section, we have described the imprint detection module in the pill recognition step shown in Figure 2 and how it is trained. In this module, we used YOLO as a text detection model. YOLO is an end-to-end model widely used in object detection [28]. In this model, when an input image is fed to multiple convolutional layers, the model classifies objects by predicting their bounding boxes in the image. The model can detect not only objects but also characters in real time. Because this model can detect texts at a rate of approximately 45 frames per second, YOLO-based text detection models have been utilized in various fields such as robotics [36], industrial automation [37], and image searching [38]. In this study, we selected YOLOv5 [29] for detecting imprinted characters on pills for real-time inference.

**Figure 3:**
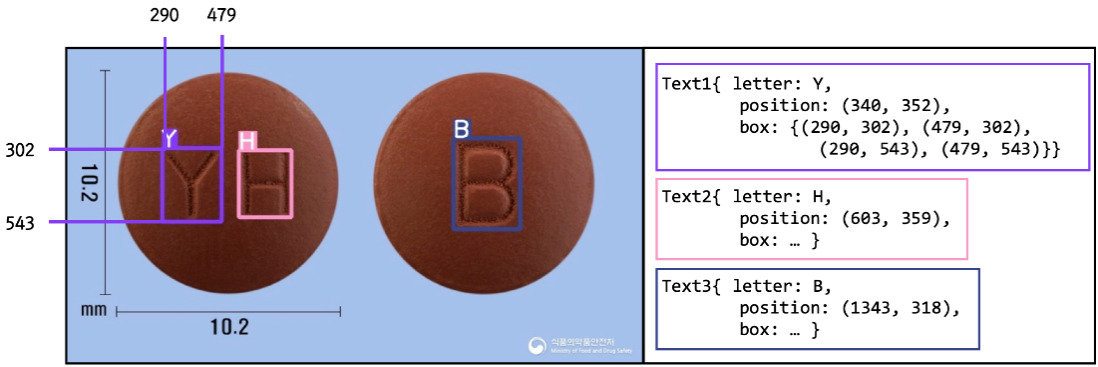
A sample of the labeled training data set for the imprint detection module is presented.

We adopted a pretrained YOLOv5 based on ImageNet [39] and fine-tuned the model using the pill images from our training data sets. Because pill images have different angles depending on pill shapes, the images are automatically rotated to place each pill horizontally using OpenCV for Python. Because there are no ground truth labels for the bounding boxes and coordinates of the characters in pill images, we annotated the labels for the bounding boxes to construct a fine-tuning data set specialized for text detection in pill images. Figure 3 shows a sample of the labeled training data set for this module.

Although character recognition using the YOLO model exhibits good accuracy, the order of characters is not considered. Furthermore, despite the high accuracy of character recognition, the network tends to confuse alphabetical characters and numbers, such as “O” and “0” or “I” and “1.” Distinguishing such characters is critical for correctly identifying pills. We noted that the misclassification of characters is a major factor leading to the poor accuracy of the previous pill identification systems that use image classification models alone. Therefore, we developed the character correction module to correct the characters extracted by the imprint detection module. We describe the correction process in detail in the imprint correction section.

#### Feature Recognition

In this section, we demonstrate the process of feature extraction using ResNet and a multitask learning method. ResNet [30] was the first model to outperform humans in the image classification competitions ImageNet Large Scale Visual Recognition Challenge in 2015 [40] and Common Objects in Context in 2015 [41]. This model was implemented for more accurate training based on the concept of residual learning. We trained ResNet-32, which consists of 32 layers, to recognize the shapes, colors, and forms of pills among the features in the input image.

We trained the model to recognize 11 shapes (*s*), 16 colors (*c*), and 2 forms (*f*), as described in the database section. ResNet-32 uses an image of a pill as an input and produces a vector output of the hidden dimension size (*h*) characterizing the pill. This vector is used to produce total feature vectors by propagating to the weight matrices *w[s]*, *w[c]*, and *w[f]* with sizes of *h* × *s*, *h* × *c*, and *h* × *f* to express the shapes, colors, and forms, respectively. For each item, 3 loss functions were calculated, and the total loss function was obtained by summing the 3 loss functions. The loss function was used to determine the error of the system prediction during training, and the model was optimized by minimizing the error. On the basis of the total loss, the weights in ResNet-32, including those of each feature, were updated.

When an image of a pill *i* produces a feature output as *p[i]* from the convolutional layer in ResNet-32, we obtain *z[i,s]=softmax(w[s]p[i])*, which contains the feature information of pill *i*, using a softmax function in the shape extraction module. The loss of the shape extraction module is determined by cross-entropy loss. The color has 16 categories, but a capsule often has more than one color; therefore, we selected 1 color for each capsule and calculated the loss in the form of cross-entropy loss. Therefore, the result corresponding to the color *j* for the pill *i* is *z[i,c]=softmax(w[c]p[i])* Finally, we divided the pill forms into tablets and capsules. The form information for the pill *i* is as follows: *z[i,f]=softmax(w[f]p[i])*. The loss functions of each feature are as follows: 



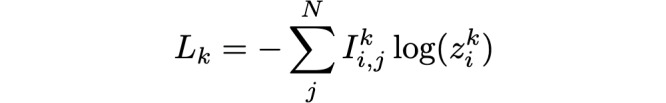



where *k* can be *s*, *c,* or *f*, which denote the shape, color, or form, respectively, and *N* denotes the number of species for each feature (11 for *s*, 16 for *c*, or 2 for *f*). Here, *I[i,j,k]* ∊(0, 1) is the ground truth label for shape, color, and form. As a result, the total loss of the shape extraction module consists of all losses multiplied by hyperparameter weights for each loss. We set all weights to 1, where the values were determined empirically.

#### Imprint Correction

We encoded the imprinted characters and features of a pill into the model by considering them as a sequence and context, respectively. Then, we trained the model to correct the characters to achieve a desired outcome. It is known that the imprints on a pill contain information regarding its ingredients, the amount of each ingredient, and the names of the medication companies. This information is important for the identification of pills. Because there is a tendency to write a series of numerical sequences on pill components and their amounts are similar to each other, the imprint calibration module can be effectively trained. The characters and their coordinates extracted by the imprint detection module (refer to the imprint detection section) are inputted into the imprint correction module. We propose a character-level RNN [31-35] with a gated recurrent unit mechanism [42] as a language model for correcting imprinted characters on pills. In this process, we propose a novel coordinate encoding technique, considering the specificity that the data are pills rather than a generic corpus. Coordinate encoding is performed to extract the position of each letter in the pill image as coordinates and enter it into the model as one piece of information for encoding it. We not only modified the imprinted characters on the pill with high accuracy but also effectively modified the order of the characters using their coordinates through a character correction module that supplements coordinate encoding techniques.

We considered an attention-based bidirectional sequence-to-sequence model [35] with a many-to-many RNN structure to correct individual characters and character orders. We were motivated by machine translation and adopted a similar system to correct imprinted characters on pills. For a character *t* with 2D coordinates (*x_t_* and *y_t_*) the input vector representation is a concatenation of the coordinate values and one-hot encoding of *t*. The representation of an input sequence is generated by the encoder and passed to the decoder. In the decoder, 1 character is generated at a time based on an attention weight that is calculated based on the character in the decoder and all the hidden states for each character in the encoder. Therefore, the conditional probability is calculated as follows: 



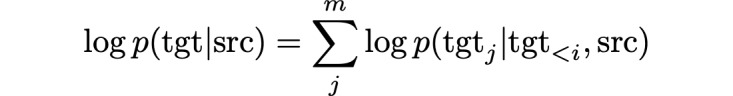



where tgt and src denote the target and source of the characters, respectively, with *m* digits of imprints. In addition, we distinguished characters on the front and back of the pills using an underscore (_). Therefore, the model should predict the exact characters and their absolute orders in the imprint. For example, if a given input extracted by the imprint detection module is “IKOIM” with coordinates for each character, then the target output is “M10_KI,” as shown in Figure 3. The features of the pills extracted by the feature recognition module can be used in the imprint correction module. We proved that the correction module improves the accuracy of pill identification, as described in the ablation study section.

### Pill Retrieval

#### Imprint Similarity Score

Our system infers pill classes based on the results of each module for a test image. We computed similarity scores between each imprint predicted from the test image and those of all the pills found in the database. After we added similarity scores from features, we obtained the top-1 and top-3 candidates by combining the similarity scores of 3 features and imprints.

We utilized the edit distance to reflect the output of the language model and the similarity scores of the imprints on pills in the database for pill retrieval. The edit distance is a measure that represents how similar 2 strings are and calculates the number of operations to be performed before one string is equal to another [43,44]. These operations include insertion, deletion, and substitution. Furthermore, the order of listing characters was also considered. We converted the edit distance into a similarity score and normalized it as follows: 



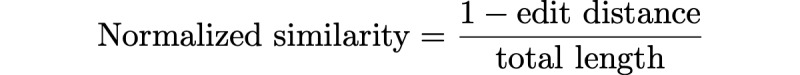



For example, if the output of the imprint correction module is “M10_KI,” then the edit distance from a target pill “M10SPC_” is 4 (3 exchanges and 1 insertion), and the normalized similarity is 6/13. Note that the underscore (_) in the imprints indicates the separation between the front and back sides. In addition, we added the information from the imprint detection module to the scores by calculating the overlap between the output of the imprint correction module (character-level language model) and the imprinted characters on the target pill, depending on the text length. We scored the overlapping characters as follows: 



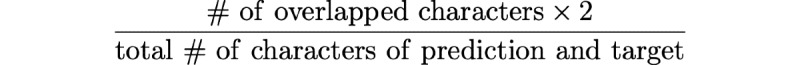



#### Feature Similarity Score

The weight of each feature (ie, shape, color, and form) was set equally in the similarity scores. However, we assumed that the similarity scores for imprints are even more crucial for identifying pills. Therefore, we assigned different weights when calculating the similarity between features and imprints. By contrast, in multilabel retrieval studies, similarity scores are mainly utilized, but probabilities are generally used. However, because our targets have different distributions for each label, unlike multilabel data, we set the similarity score weights for the 3 feature types to 1/3 only for the exact match of the labels. For example, if the output of the model is [square, orange, tablet], then a pill with [square, pink, tablet] has 2/3 points, a pill with [triangle, orange, capsule] has 1/3 points, and a pill with [round, yellow, capsule] has 0 points.

Table 1 presents an example of the calculation of the similarity scores for each pill in the database. Imprint similarity scores are the last 2 terms written in italics under scores, which range from 0 to 1. One is based on the edit distance, and the other is based on the number of overlapping characters between the output of the character-level language model and the target character sequences. According to Table 1, even if the other features are the same as the source, as is the case for target 3, the score is lower than if the imprinted characters are more similar. Because the correct pill corresponding to the source is target 1, we can conclude that accurate imprint recognition is the most important aspect of pill identification.

**Table 1:** Example of similarity scores for 3 target pills in the database with given source information^a^ extracted by the proposed system.

	Characteristics of target pills	Scores
Target 1	[square, pink, tablet, M10_KI]	1/3 + 0 + 1/3 + *1* + *1* = 2.67
Target 2	[square, pink, tablet, M10_Kb]	1/3 + 0 + 1/3 + *10/12* + *10/12* = 2.33
Target 3	[square, orange, tablet, M10SPC_]	1/3 + 1/3 + 1/3 + *6/13* + *8/13* = 2.08

^a^Source: [square, orange, tablet, M10_KI].

## Results

### Overall Experiments

We evaluated the developed pill identification system in terms of the accuracy of top-1 and top-3 similarity score candidates. For convenience, we defined the probability that the answer is in the top-1 and top-3 candidates as top-1 and top-3 accuracy. Note that previous works considered top-1 and top-5 candidates [22], but we chose top-3 candidates instead of top-5 candidates because our system has similar accuracy for top-3 and top-5 candidates. First, we trained our model on the reference images of 38.99% (8000/20,517) of kinds of pills in the MFDS database. Experiments to be evaluated with a trained system attempt to answer the following questions: (1) Can the model predict new species of pills that have not been used for training well? (2) Can the model be utilized for different types of data, not just the type of data it was trained on? Can the model predict new species of pills that have not been used in training? (3) Can the model predict images of consumer ratings in addition to stereotyped images? and (4) Does this contribute to the performance of our proposed language model for pill identification? We have proceeded with an evaluation to answer questions 1 to 3 in the model evaluation section and discussed question 4 in the ablation section.

### Model Evaluation

**Figure 4:**
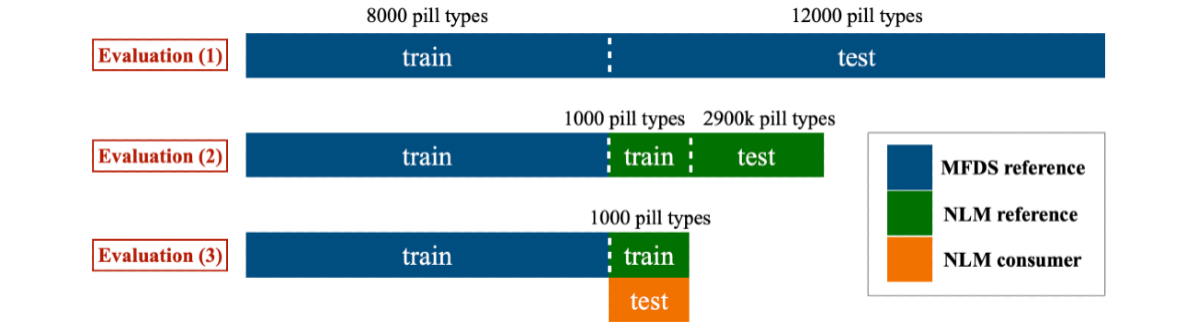
Training and test data sets for 3 evaluation experiments are shown. Deep blue bars, green bars, and an orange bar indicate the Ministry of Food and Drug Safety (MFDS) reference images, National Library of Medicine (NLM) reference images, and NLM consumer images, respectively.

We trained the system on the 38.99% (8000/20,517) of reference images from the MFDS database via oversampling to represent less frequently appearing colors, shapes, and forms (Multimedia Appendix 1). We evaluated the system using 61.01% (12,517/20,517) of images from the MFDS database as reference images and 74.27% (2887/3887) of images from the NLM database. Note that we fine-tuned the system on 25.73% (1000/3887) of reference images from the NLM database before evaluating the NLM images. Furthermore, after fine-tuning the system on 25.73% (1000/3887) of reference images from the NLM database, we evaluated it on 25.73% (1000/3887) of consumer images from the NLM database containing the same pills. In this case, simple image preprocessing such as concatenating pills horizontally was automatically applied to the consumer images. Figure 4 illustrates the training, fine-tuning, and evaluation processes.

During training, we experimentally established suitable hyperparameters according to each module. We implemented our system and baseline using PyTorch and trained our system on a single machine equipped with an AMD 12-core processor, 128 GB of RAM, and NVIDIA GeForce RTX 3090 with 11 GB of RAM for 2.3 hours. The imprint detection module was trained using a stochastic gradient descent optimizer with a learning rate of 10^-2^, 3 warm-up epochs, and a batch size of 16. The feature recognition module was trained for 100 epochs for 0.2 hours using a stochastic gradient descent optimizer with a learning rate of 0.1, weight decay of 10^-4^ and batch size of 10 for 1.8 hours. Finally, the imprint correction module was trained for 100 epochs using an Adam optimizer with a learning rate of 10^-3^ and batch size of 50 for 0.3 hours, where the embedding size and size of the hidden layer in the language model were 45 and 256, respectively.

We first evaluated 61.01% (12,517/20,517) of sample pill images from the MFDS database and retrieved the pills from the database by computing the similarity scores between each evaluation image and all pill images in the test data set. The second and third columns in Table 2 present the evaluation results in terms of the top-1 and top-3 candidate accuracies on the NLM database. We compared our system’s performance to that of a system using ResNet-50 (baseline) [22]. The top-1 accuracy on the MFDS samples was 85.65%, and the top-3 accuracy was 92.35%. It is clearly possible for our system to identify pills that are not used in training because of the role of the retrieval system. By contrast, the baseline system performs classification, rather than retrieval, based on the labels of each pill. Therefore, the baseline systems could not identify pills that were not used in training.

The results for 74.27% (2887/3887) of NLM reference images show that the top-1 accuracy is 74.46%, and the top-3 accuracy is 88.7%. This indicates that the proposed system can identify pills in 2 different databases with fine-tuning. This demonstrates the structural advantages of our system because it is feasible to apply another database by simply including a small number of sample images without making any structural changes to the system. Furthermore, this result implies that the proposed system does not rely heavily on a particular database.

Next, we trained the model on only 25.73% (1000/3887) of reference images from the NLM database to evaluate the applicability of the model and compared it with the baseline on 1000 consumer images. We preprocessed the images by horizontally concatenating the front and back sides of the pills. Our system exhibited a top-1 accuracy of 78% and top-3 accuracy of 89.1%, which outperformed the baseline model that resulted in 76.9% top-1 accuracy and 81.1% top-3 accuracy. In addition, we confirmed that our system can identify a pill in real time by taking approximately 0.789 seconds in pill evaluation for consumer images.

**Table 2:** Accuracies of the proposed system in terms of the top result on 3 evaluations compared with the baseline [22] are shown. Each result indicates the evaluation on the Ministry of Food and Drug Safety (MFDS) reference images, National Library of Medicine (NLM) reference images, and NLM consumer images. Top 1 and top 3 denote the probability that the answer is in the top-1 and top-3 candidates.

	MFDS database		NLM database		NLM consumer	
	Top 1 (%)	Top 3 (%)	Top 1 (%)	Top 3 (%)	Top 1 (%)	Top 3 (%)
Baseline system	—^a^	—	—	—	76.9	81.1
Our system	85.65	92.35	74.46	88.70	78	89.1

^a^Not available.

### Ablation Study

We evaluated the influence of the imprint correction module by ablating it from the imprint detection and feature recognition modules. This analysis aimed to examine our hypothesis that the imprint correction module is the most important for improving the accuracy of the system. We determined that the system without an imprint correction module struggled to identify pills in cases where the imprinted character string was long. In such cases, imprints are difficult to identify because of the nonlinear arrangement of imprinted characters or confusion between alphabetical letters and numbers. Table 3 demonstrates that the accuracy increased by 3.3% to 11.3% when the imprint correction module was added. These results prove our hypothesis that many errors occur in the absence of additional treatment for characters.

We used another ablation study on the similarity score to demonstrate the importance of imprints in pill images. Table 4 shows the top-1 accuracy and top-3 accuracy of the proposed system when each characteristic (shape, color, form, and imprint) was missing in the similarity score. When the similarity score of imprints was omitted, the system showed a top-1 accuracy of 0%, indicating that the pill cannot be identified. Even among the top-3 candidates, the probability was only 0.05%.

**Table 3:** The accuracies for the Ministry of Food and Drug Safety (MFDS) data set (left) and the National Library of Medicine (NLM) data set (right) are shown. The case without the imprint correction module is in the upper row (without language model [LM]), and the case with it is in the lower row (with LM).

	MFDS database		NLM database	
	Top 1 (%)	Top 3 (%)	Top 1 (%)	Top 3 (%)
Without LM	74.4	83.9	71.46	84.68
With LM	85.7	92.4	74.76	88.7

**Table 4:** The accuracies by ablating each characteristic (shape, color, form, and imprint) of pills in the similarity score for the Ministry of Food and Drug Safety data set are shown. “None” denotes our proposed system considering all characteristics in the similarity score.

Ablated characteristic	Top 1 (%)	Top 3 (%)
None	85.65	92.35
Shape	83.88	91.59
Color	81.08	89.74
Form	85.94	92.29
Imprint	0	0.05

## Discussion

### Principal Findings

This work demonstrates that incorporating language models into a deep learning–based pill identification system significantly improves the accuracy of the system and helps the system generalize to other data.

We verified the generalization and robustness of our system by using different pill types for evaluation from those used in training. The experimental results demonstrated that our system could identify pills that were not used in training. Therefore, our system can identify newly enrolled pills without additional training. In addition, we evaluated 74.27% (2887/3887) of pill samples from the NLM database of the United States and 61.01% (12,517/20,517) of pill samples from the MFDS database of South Korea. The experimental results demonstrate that our model does not rely on a particular database. Furthermore, the proposed system can reduce database dependence and achieve high performance on 2 different databases because it learns the characteristics of pills by segmenting them into 3 features (color, shape, and forms) and imprinted characters and measures the similarities of each predicted characteristic to characteristics of target pills. We conducted experiments on identifying consumer images while training our system on reference images for comparison with the baseline and a discussion of the system’s applicability. Because the consumer images in the NLM database have various lighting conditions, the pill identification task was more challenging. In this evaluation, our model outperformed the baseline, as shown in Table 2.

The ablation study section determined that the imprint correction module can significantly improve identification performance. Table 3 presents the results with and without the imprint correction module based on the characters and their coordinates extracted by the imprint detection module. An RNN, which exhibits good performance for deep learning–based machine translation, is used for correcting imprinted characters as a character-level language model. In the absence of this module, the system uses the initial results from imprint detection to calculate similarity scores. The results in Table 3 prove our hypothesis that pills can be identified more accurately by implementing an imprint correction module in the form of a language model. Pills are difficult to identify based solely on their shape, color, and form. Rather, it is important for pill identification to accurately detect imprinted characters because imprints contain information regarding pill ingredients, their amounts, and the pill manufacturer. To the best of our knowledge, ours is the first attempt to extract each character and its coordinates separately and then correct them using a language model in the pill identification system. In addition, rather than simply predicting and classifying the labels of the pills, the similarity scores with the pills in the database were calculated by considering different characteristics and assigning larger weight values to the imprints. Moreover, in another ablation study, as shown in Table 4, imprint was shown to have a crucial role in identifying pills. By contrast, form makes little contribution to the identification of pills.

### Comparison With Prior Work

Table 2 presents the results of a state-of-the-art system [22] using ResNet-50 and a classification system and compares them with those of our system obtained in the same environment. The former systems [13,17-23,25] identify pills by extracting a single feature from the image of a pill and then performing classification based on class labels via a softmax function. By contrast, our system distinguishes the features of the pill by color, shape, form, and imprint and then retrieves the matching pills from a database. Our system detects characters and their coordinates and then corrects the result more precisely, unlike previous studies that did not consider imprints carefully. We used ResNet, which is also used in the baseline, but considered fewer layers than the baseline (50 vs 32). We added separate modules designed to extract features and imprints to establish a much more specific and accurate identification process. We found that the system proposed by Zeng et al [21] used the same data set as that used by Larios Delgado et al [22]. However, we chose the system from Larios Delgado et al [22] as the baseline because its performance was superior. We used 2 different databases to demonstrate the effectiveness of the proposed system. We trained the model with 38.99% (8000/20,517) of pill samples from the database provided by the MFDS in South Korea for our first 2 experiments. The evaluation was conducted on 61.01% (12,517/20,517) of MFDS samples that were completely different from those used for training. There is a system that achieved excellent pill identification results, but only 500 pills were used for both training and evaluation [13]. The biggest difference between our system and previous systems is that our system can identify pills that are not used in training, whereas previous systems could not identify new pills. This is because we constructed our system as a retrieval system, rather than a classification system.

### Strengths and Limitations

We introduce 2 strengths of the proposed system. First, the proposed system can identify new types of pills that have not been used in learning. Even if the new drug is approved and the database is updated, further learning of the system is not required. Second, the proposed system has a high generalization capability for the database. We pretrained the system with data from the MFDS and evaluated it by fine-tuning it with much less NLM data to confirm that the system shows promising results.

However, our system has 3 limitations. First, we used only capital letters except for “mg” (milligram) as the imprinted letters of the pill. Pills with lowercase letters were difficult to learn because of the small amount of data and, consequently, were not used in training. Symbols were not used in training for the same reason. We will compensate these limitations by collecting more data from future studies. Second, we observed that the experimental results for the NLM data demonstrated slightly lower performance than the results for the MFDS data. This is largely attributed to the fact that the system is pretrained on large amounts of the MFDS data and fine-tuned on small amounts of NLM data. To maintain the knowledge from large data and exhibit stable performance in various databases, we will later attempt to introduce transfer learning techniques via multitask learning [45] or adapter [46]. Third, when extracting pills from the images, we extracted them separately from the background using an algorithm without applying a deep learning–based segmentation model. From the image of the database we used, the pill shape was extracted with high accuracy just by extracting it with an algorithm. However, because various backgrounds, shading, and some broken pills may exist in real-world images, the latest model based on deep learning is expected to be helpful. In the future, it is expected that the system can be improved by using photos of actual drugs and applying deep learning–based sedation through collaboration with research teams in the pharmaceutical field.

### Conclusions

In this study, we hypothesized that the most important information in a pill image lies in imprinted characters and proposed a pill identification system utilizing imprinted characters. Unlike most pill identification systems that use only models specialized for image classification, we implemented a character-level language model to achieve high-accuracy pill recognition. To evaluate the robustness and generalizability of the proposed system, it was evaluated using 2 different pill databases in South Korea and the United States. Unlike the baseline systems, our system can identify pills that are not contained in the training data set. In addition, we conducted pill identification experiments on consumer images of pills that were only seen once during training to compare our system with the baseline. The baseline, which utilizes CNNs, has achieved outstanding performance among the existing systems. However, our system achieved a higher accuracy than the baseline. Moreover, our system shows high accuracy while taking less than 1 second to identify the pill, so it is expected to be utilized in devices such as previously proposed wearable devices [23] or mobile apps [19,21]. Therefore, it is hoped that the study on AI-based pill identification systems can be advanced based on the foundation laid by our study.

## Appendix

Multimedia Appendix 1 Distribution in training data set of the Ministry of Food and Drug Safety.

## Abbreviations

AI: artificial intelligence
CNN: convolutional neural network
MFDS: Ministry of Food and Drug Safety
NLM: National Library of Medicine
OECD: Organisation for Economic Co-operation and Development
ResNet: Residual Network
RNN: recurrent neural network
YOLO: You Only Look Once

## Data Availability

The data sets generated during and analyzed during this study are available from the corresponding author upon reasonable request. The code is available from the corresponding author upon reasonable request.
