# Supplementary figures and images for: An Accurate Deep Learning–Based System for Automatic Pill Identification: Model Development and Validation
Source: J Med Internet Res. 2023 Jan 13;25:e41043. doi: 10.2196/41043 (PMC9883737; doi:10.2196/41043)

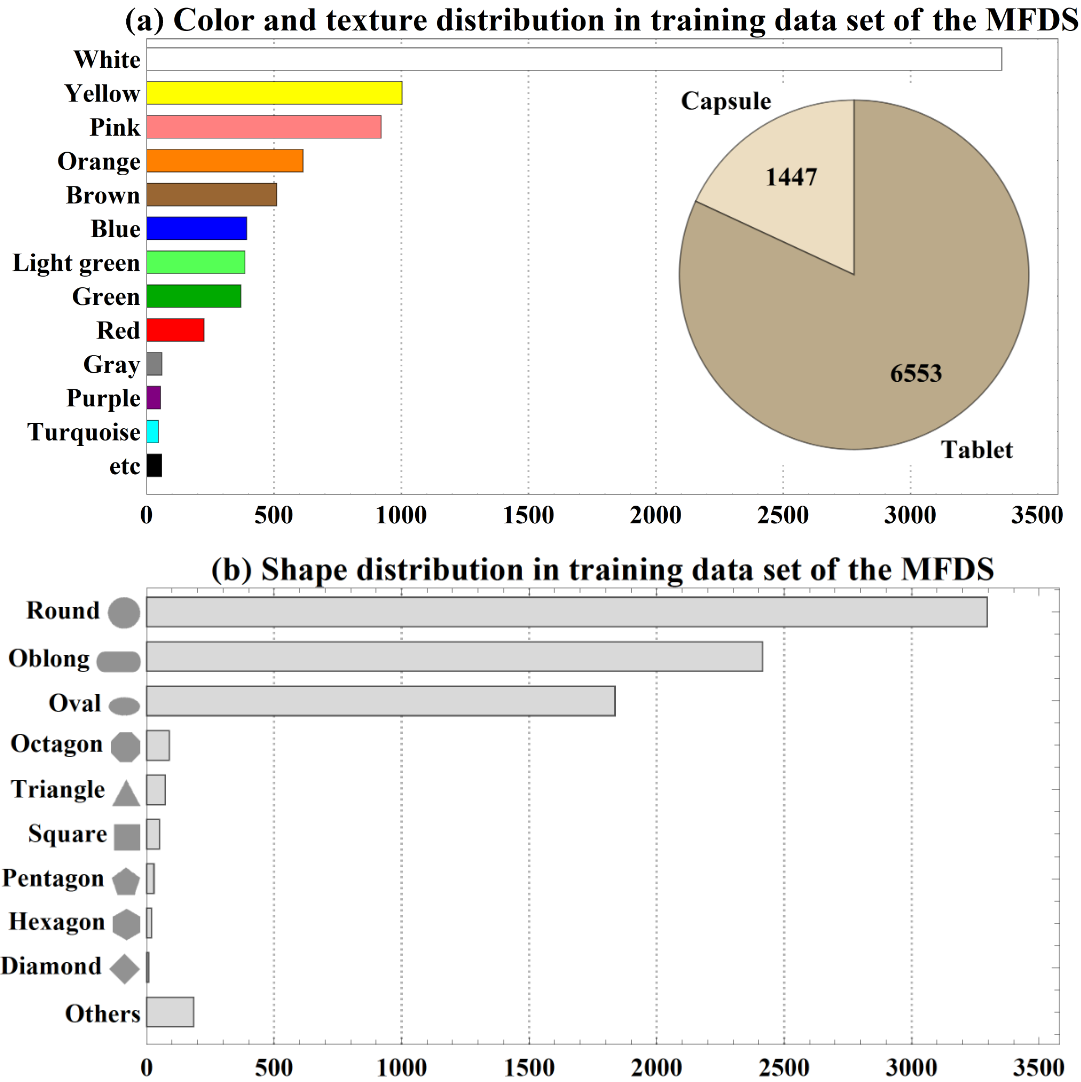

Supplement: Multimedia Appendix 1 [file jmir_v25i1e41043_app1.png]
